# Interleukin-21 induces the differentiation of human umbilical cord blood CD34^-^lineage^- ^cells into pseudomature lytic NK cells

**DOI:** 10.1186/1471-2172-10-46

**Published:** 2009-08-27

**Authors:** Giuseppina Bonanno, Andrea Mariotti, Annabella Procoli, Maria Corallo, Giovanni Scambia, Luca Pierelli, Sergio Rutella

**Affiliations:** 1 Department of Gynaecology, Catholic University Medical School, Rome, Italy; 2 Department of Haematology and Cell Therapy, Azienda Ospedaliera "San Camillo-Forlanini", Rome, Italy; 3 Department of Haematology, Laboratory of Immunology, Catholic University Medical School, Rome, Italy; 4 IRCCS San Raffaele Pisana, Rome, Italy

## Abstract

**Background:**

Umbilical cord blood (UCB) is enriched with transplantable CD34^+ ^cells. In addition to CD34-expressing haematopoietic stem cells (HSC), human UCB contains a rare population of CD34^-^lineage^- ^cells endowed with the ability to differentiate along the T/NK pathway in response to interleukin (IL)-15 and a stromal cell support. IL-21 is a crucial regulator of NK cell function, whose influence on IL-15-induced differentiation of CD34^-^lineage^- ^cells has not been investigated previously. The present study was designed and conducted to address whether IL-21 might replace the stromal cell requirements and foster the IL-15-induced NK differentiation of human UCB CD34^-^lineage^- ^cells.

**Results:**

CD34^-^lineage^- ^cells were maintained in liquid culture with Flt3-L and SCF, with the addition of IL-15 and IL-21, either alone or in combination. Cultures were established in the absence of feeder cells or serum supplementation. Cytokine-treated cells were used to evaluate cell surface phenotype, expression of molecular determinants of lymphoid/NK cell differentiation, secretion of IFN-γ, GM-CSF, TNF-α and CCL3/MIP-1α, and cytolytic activity against NK-sensitive tumour cell targets. CD34^-^lineage^- ^cells proliferated vigorously in response to IL-15 and IL-21 but not to IL-21 alone, and up-regulated phosphorylated Stat1 and Stat3 proteins. CD34^-^lineage^- ^cells expanded by IL-21 in combination with IL-15 acquired lymphoid morphology and killer-cell immunoglobulin-like receptor (KIR)^-^CD56^+^CD16^-/+ ^phenotype, consistent with pseudo-mature NK cells. IL-21/IL-15-differentiated cells expressed high levels of mRNA for Bcl-2, GATA-3 and Id2, a master switch required for NK-cell development, and harboured un-rearranged TCRγ genes. From a functional standpoint, IL-21/IL-15-treated cells secreted copious amounts of IFN-γ, GM-CSF and CCL3/MIP-1α, and expressed cell surface CD107a upon contact with NK-sensitive tumour targets, a measure of exocytosis of NK secretory granules.

**Conclusion:**

This study underpins a novel role for IL-21 in the differentiation of pseudo-mature lytic NK cells in a synergistic context with IL-15, and identifies a potential strategy to expand functional NK cells for immunotherapy.

## Background

Umbilical cord blood (UCB) is increasingly used as an alternative source of transplantable CD34^+ ^haematopoietic stem cells (HSC) for neoplastic and non-neoplastic diseases [1]. The function of CD34 antigen on human HSC is poorly understood. It has been shown that small interfering RNA-mediated gene silencing of CD34 on human HSC from UCB favours granulocytic and megakaryocytic development at the expense of erythroid commitment, thus shedding light into the potential functional role of this molecule during haematopoietic differentiation [2]. In recent years, HSC with a CD34^- ^phenotype have been identified in human UCB, unravelling a hitherto unrecognized complexity within the haematopoietic hierarchy [3,4]. Previously, we characterized a rare subpopulation of human UCB CD34^-^CD133^-^CD7^-^lineage^- ^cells capable of differentiating both into CD34^+^CD133^+ ^HSC in response to stem cell factor (SCF), and into NK/lymphoid progenitors if supported by interleukin (IL)-15 and stromal cells engineered to release human granulocyte colony-stimulating factor (G-CSF) and IL-3 [5]. In line with this, UCB-derived mesenchymal stem cells have been used to support NK cell expansion induced by the combination of IL-2, IL-3, IL-15 and Flt3-L [6]. Similarly, Wharton's jelly cells may serve as feeder cells to expand UCB-derived CD34^+ ^HSC in a potentially clinically applicable culture system [7]. It should be pointed out that mesenchymal stem cells may activate allogeneic T cells during *in vitro *HSC expansion [8], suggesting need for feeder cell-free culture systems that may support HSC expansion in the absence of untoward effects on other cell types.

IL-21 is a four-helix bundle cytokine released by activated CD4^+ ^T cells and by NKT cells [9]. IL-21 signals through a heterodimeric receptor comprising the IL-21 receptor and the common γc of the IL-2 receptor family. IL-21 affects the differentiation and proliferation of NK cells together with IL-2 and IL-15, and is involved in the differentiation of T-helper 17 (Th17) cells, a recently identified subset of CD4^+ ^T cells that produce IL-17A, IL-17F and IL-22 and promote inflammatory and autoimmune conditions [10]. In addition, IL-21 suppresses the differentiation of FoxP3-expressing regulatory T cells, leading to enhanced cytotoxic T lymphocyte (CTL) expansion and activity [11]. Finally, IL-21 is a key regulator of antibody responses against foreign antigens [12], suggesting that IL-21 may be a master orchestrator of the T-cell-dependent adaptive immune response.

In mice, IL-21 acts in concert with IL-15 to boost the proliferation of both memory and naïve CD8^+ ^T cells and to foster the *in vitro *release of IFN-γ [13]. Interestingly, IL-21 selectively enhances the effector functions of IL-15-activated murine NK cells, further underpinning the importance of functional interactions between the two cytokines, and mediates potent *in vivo *anti-tumour responses [14]. When provided to serum-replenished cultures of UCB CD34^+^lineage^- ^cells, IL-21 in combination with IL-15, IL-7, Flt3-L and SCF reportedly induces an accelerated NK cell maturation [15]. Furthermore, IL-21 cooperates with hydrocortisone, IL-15 and Flt3-L in supporting the expansion of NK cells from UCB CD34^+ ^cells [16]. However, the contribution of IL-21, if any, to the NK cell differentiation of CD34^-^lineage^- ^cells has not been investigated. It is also unknown whether CD34^-^lineage^- ^cells stimulated with IL-21 may give rise to a qualitatively different NK population when compared to CD34^+ ^HSC.

The present study aimed to address whether IL-21 might replace the stromal cell requirements and foster the IL-15-induced NK differentiation of human UCB CD34^-^lineage^- ^cells.

## Results

### Isolation and phenotypic characterisation of UCB CD34^-^lineage^- ^cells

**Figure 1:**
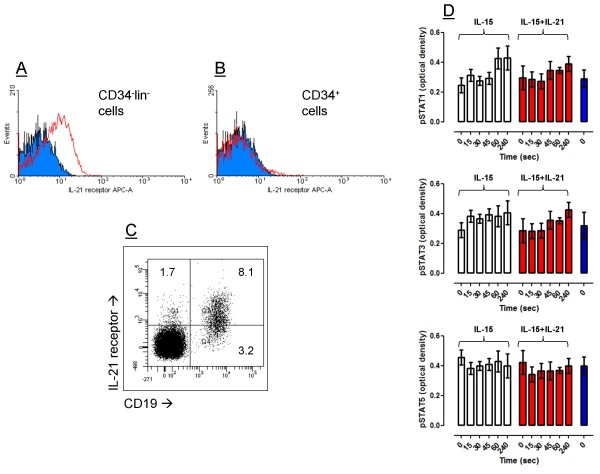
**Expression of IL-21 receptor on CD34^-^lineage^- ^cells and Stat protein activation by IL-21**. **Panel A**: Freshly isolated CD34^-^lineage^- ^cells were incubated with APC-conjugated anti-IL-21 receptor mAb, followed by flow cytometry analysis. Isotype-matched, irrelevant mAb were used to control for background staining (cyan histogram). One representative experiment out of 4 with similar results is shown. **Panel B**: CD34^+ ^cells were separated from UCB samples using the MACS^® ^system, as detailed in Material and Methods. Cells were labelled with anti-IL-21 receptor mAb prior to flow cytometry analysis. Isotype-matched, irrelevant mAb were used to control for background staining (cyan histogram). One representative experiment out of 4 with similar results is shown. **Panel C**: Mononuclear cells from the peripheral blood of healthy blood donors were labelled with anti-IL-21 receptor mAb prior to flow cytometry analysis. Markers were set according to the proper isotypic control. One representative experiment out of 4 with similar results is shown. **Panel D**: Stat protein phosphorylation was measured after provision of either 50 ng/ml IL-15 (white columns) or 50 ng/ml IL-15 + 20 ng/ml IL-21 (red columns) to freshly isolated CD34^-^lineage^- ^cells. Bars depict the mean and standard deviation recorded in 4 independent experiments performed in triplicate. The blue bars depict the level of phosphorylated Stat1 (Tyr701), Stat3 (Tyr705) and Stat5 (Tyr694) in freshly isolated peripheral blood mononuclear cells, used as control.

Freshly isolated CD34^-^lineage^- ^cells stained negatively for stem cell-associated (CD34, CD133) and NK/lymphoid surface antigens (CD7, CD56, CD16, CD3, TCRαβ; data not shown), and comprised 0.22% on average of UCB mononuclear cells (samples analyzed = 11). The aforementioned frequency of CD34^- ^cells is in good agreement with that reported by others [3]. CD34^-^lineage^- ^cells expressed low levels of IL-21 receptor protein (Figure 1A). The expression of IL-21 receptor in control samples, e.g., UCB CD34^+ ^HSC and human peripheral blood B cells, is shown in Figure 1B and Figure 1C, respectively. Following the observation that Stat1/Stat3 deficient CD8^+ ^T cells manifest decreased responsiveness to IL-21 [17], we attempted to determine whether IL-21 induced Stat protein phosphorylation in CD34^-^lineage^- ^cells. When freshly isolated CD34^-^lineage^- ^cells were stimulated with 20 ng/ml IL-21 for 4 hours, both phosphorylated Stat1 and phosphorylated Stat3 were induced, whereas phosphorylated Stat5 levels remained unaffected (Figure 1D).

**Figure 2:**
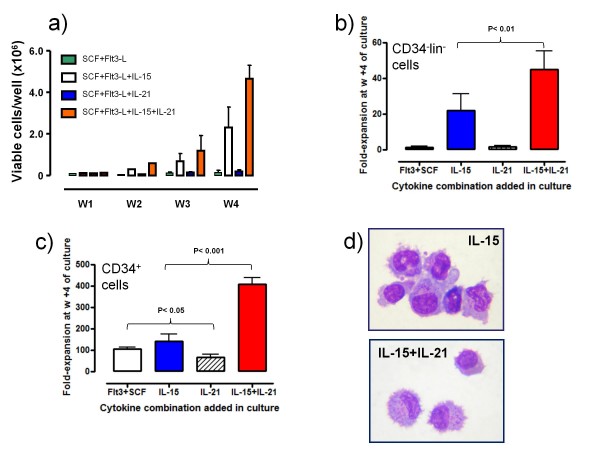
**Expansion of CD34^-^lineage^- ^cells in response to IL-15 plus IL-21**. CD34^-^lineage^- ^cells were maintained in liquid cultures with Flt3-L and SCF, in the presence of either IL-15, or IL-21 or the combination of both cytokines for up to 4 weeks. In order to monitor cell expansion in response to cytokine stimulation, cells were harvested weekly and counted. CD34^+ ^cells isolated from the same UCB samples were used in control experiments. **Panel A**: Cultures were established with CD34^-^lineage^- ^cells. The number of viable cells was counted using trypan blue exclusion. The bars depict mean and standard deviation recorded in 11 independent experiments performed with 11 different UCB samples. **Panel B**: Fold expansion of the CD34^-^lineage^- ^cells after 4 weeks of culture with either of the cytokine combinations. The bars depict mean and standard deviation recorded in 11 independent experiments performed with 11 different UCB samples. **Panel C**: Fold expansion of UCB CD34^+ ^cells after 4 weeks of culture with either of the cytokine combinations. The bars depict mean and standard deviation recorded in 5 independent experiments performed with 5 different UCB samples. **Panel D**: After 4 weeks of culture with IL-21 and IL-15, CD34^-^lineage^- ^cells were harvested, stained with May-Grünwald-Giemsa and visualized under an AX70 optical microscope (Olympus, Tokyo, Japan) equipped with 100×/1.25 NA objective lens. Image acquisition was performed with an Optronics digital camera (Olympus) and ImagePro Plus software (Media Cybernetics, Silver Spring, MD).

CD34^-^lineage^- ^cells were next maintained with SCF and Flt3-L for up to 4 weeks, and IL-15 or IL-21 or the combination of both cytokines was supplemented to SCF+Flt3-L-containing cultures as indicated in the Figure legends. Either IL-15 alone or, even more dramatically, the combination of IL-15 and IL-21 activated the proliferation of CD34^-^lineage^- ^cells (Figure 2A). After culturing with Flt3-L and SCF supplemented with the combination of IL-15 and IL-21, CD34^-^lineage^- ^cells were expanded by 42.5-fold on average (Figure 2B), in contrast with the effects of Flt3-L and SCF either alone or supplemented with IL-15 or IL-21 individually. Control cultures were comprised of UCB CD34^+ ^cells exposed to the same cytokine combinations as the CD34^-^lineage^- ^cells. CD34^+ ^cells were incapable of responding to IL-21 alone (data not shown), in accordance with previous reports demonstrating lack of IL-21 receptor expression on freshly isolated CD34^+ ^cells [16]. As illustrated in Figure 2c, CD34^+ ^cells expanded vigorously in response to Flt3-L and SCF, either alone or supplemented with IL-15. However, proliferation was significantly increased in CD34^+ ^cell cultures containing both IL-15 and IL-21, indicating synergistic effects of the two γc signalling cytokines. Interestingly, CD34^+ ^cell expansion promoted by Flt3-L and SCF was constrained by the addition of IL-21 (Figure 2C). Not unexpectedly, CD34^-^lineage^- ^cells nurtured with IL-15 either alone or in combination with IL-21 acquired a peculiar lymphoid morphology with eccentric nuclei and heavy cytoplasmic granules (Figure 2D).

**Figure 3:**
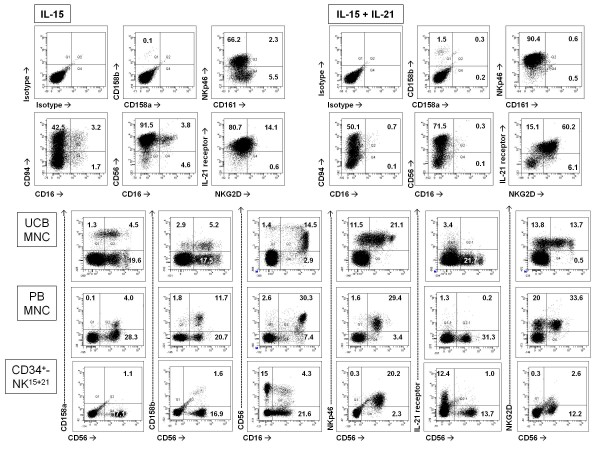
**Phenotypic features of cytokine-differentiated CD34^-^lineage^- ^cells**. After 4 weeks of culture with either IL-15 or the combination of IL-15 and IL-21, CD34^-^lineage^- ^cells were harvested, stained with a panel of mAb directed against informative differentiation/maturation antigens and used for flow cytometry analysis. Fluorochrome-conjugated, isotype-matched mAb from the same manufacturers were used to measure background fluorescence. Mononuclear cells (MNC) from UCB and peripheral blood (PB) samples as well as NK cells matured *in vitro *with Flt3-L, SCF, IL-15 and IL-21 from UCB CD34^+ ^cells (CD34^+^-NK^15+21^) were used as control for antigen expression. The percentage of cells staining positively for each given antigen is indicated in the dot plots. One representative experiment out of 8 with similar results is shown.

We investigated the expression of an array of potentially informative activation/differentiation antigens on cytokine-modulated CD34^-^lineage^- ^cells. The results of immunophenotypic studies are summarised in Table 1. As depicted in Figure 3, neither CD158a nor CD158b antigens could be detected on IL-15-differentiated CD34^-^lineage^- ^cells. Consistent with the surface phenotype assigned to pseudo-mature NK cells [18], a minute percentage (< 3%) of IL-15+IL-21-differentiated cells expressed CD158a and CD158b, indicating that killer-cell immunoglobulin-like receptors (KIR) were not induced by either cytokine on CD34^-^lineage^- ^cells. Whereas CD56 was highly expressed by CD34^-^lineage^- ^cells cultured with either IL-15 alone or the combination of IL-15 and IL-21, a minor subset of CD56^+^CD16^+ ^cells could be consistently detected both in cultures maintained with IL-15 alone and in those nurtured with IL-15+IL-21. NKp46, a NK triggering receptor [19], was found on significantly higher percentages of cells cultured with IL-15 and IL-21 compared with those maintained with IL-15 alone. Conversely, the CD94-NKG2A inhibitory receptor complex was expressed by comparable percentages of IL-15-differentiated and IL-21/IL-15-differentiated CD34^-^lineage^- ^cells (Figure 3). Finally, NKG2D, a powerful activating receptor involved in the regulation of immune responses during infection, cancer and autoimmunity [20], was preferentially detected on CD34^-^lineage^- ^cells cultured with IL-15 and IL-21 (Figure 3). The immunophenotypic characteristics of NK cells differentiated *in vitro *from UCB CD34^+ ^cells and those of NK cells from UCB and peripheral blood are depicted in Figure 3. When compared with CD34-derived NK cells and with pre-formed NK cells, CD34^-^lineage^- ^cells emerging from IL-15+IL-21-containing cultures expressed very low levels of CD16 and KIR, but high levels of CD56, NKG2D and IL-21 receptor. Because NKG2D expression may be negatively regulated by TGF-β [21], we added exogenous TGF-β at 5 ng/ml to CD34^-^lineage^- ^cells concomitant with their exposure to IL-15 and IL-21. Under these conditions, less than 5% of developing NK cells stained positively for NKG2D (data not shown), suggesting that NKG2D expression induced by IL-15 and IL-21 was sensitive to cytokine regulation. Insufficient numbers of cells for detailed phenotypic analyses were recovered from cultures performed with Flt3-L and SCF either alone or supplemented with IL-21, thus precluding a thorough evaluation of the expression pattern of NK cell-associated antigens on those cell populations (data not shown).

**Table 1:** Surface phenotype of cytokine-differentiated and freshly isolated NK cells.

**Cell surface antigen**	**UCB MNC**	**PB MNC**	**CD34^+^-derived NK cells** **(15+21)**	**CD34^-^lineage^- ^derived** **NK cells** **(IL-15)**	**CD34^-^lineage^- ^derived** **NK cells** **(15+21)**
CD56^+^	23 ± 5	35.4 ± 9	22.9 ± 5	94 ± 2	92 ± 2
CD56^+^16^+^	14.5 ± 7	29.5 ± 5	6.5 ± 2	4 ± 1	0.6 ± 0.2
CD56^+^94^+^	20.9 ± 4	26.7 ± 7	17.6 ± 4	47.5 ± 8	52 ± 5
CD56^+^NKp46^+^	22.3 ± 5	28.6 ± 4	21.1 ± 4	65.5 ± 8	91.5 ± 1
CD56^+^NKG2D^+^	15.4 ± 2	31.4 ± 9	5.0 ± 0.8	16.5 ± 4	68.5 ± 5
CD56^+^158a^+^	4.6 ± 1	4.5 ± 1.5	0.6 ± 0.5	0.01 ± 0.01	0.5 ± 0.2
CD56^+^158b^+^	5.9 ± 1	11.5 ± 6	1.2 ± 1	0.1 ± 0.1	1.8 ± 0.5
CD56^+^161^+^	23.3 ± 6	32.2 ± 7	1.5 ± 0.5	8.3 ± 2	2.1 ± 0.5
CD56^+^244^+^	16.9 ± 6	30.8 ± 4	19.8 ± 5	89.6 ± 5	81.7 ± 2
IL-21R^+^	3.6 ± 0.9	2.2 ± 1	12.9 ± 6	94.2 ± 2	76.5 ± 3

### Molecular profile of IL-21-differentiated CD34^-^lineage^- ^cells

**Figure 4:**
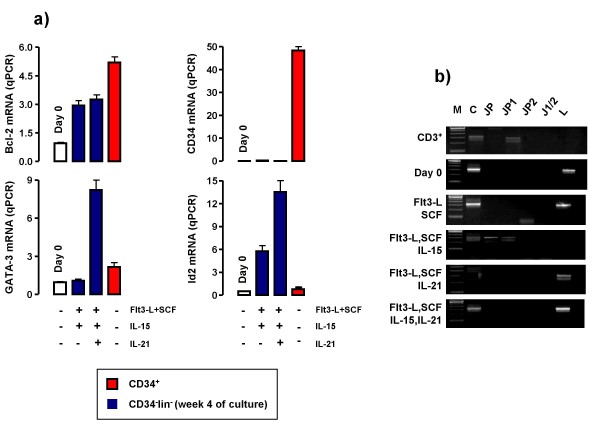
**Molecular profile of CD34^-^lineage^- ^cells differentiated with IL-15 plus IL-21**. **Panel A**: Molecular features of cytokine-differentiated CD34^-^lineage^- ^cells. The expression of mRNA specific for the NK-associated transcription factor Id2 and for Bcl-2 and GATA-3 was investigated by quantitative PCR (qPCR) in freshly isolated (day 0) and cytokine-differentiated CD34^-^lineage^- ^cells (week +4), as previously detailed [5]. CD34^+ ^cells from the same UCB samples were used as control. **Panel B**: TCRγ rearrangement status after *in vitro *exposure to IL-15 plus IL-21. The analysis of TCRγ rearrangement status was performed as detailed in Materials and Methods. Peripheral blood CD3^+ ^T cells from healthy donors were used as control for TCRγ rearrangement status. CD34^-^lineage^- ^cells cultured with IL-15 and IL-21 harboured un-rearranged TCRγ genes, similar to freshly isolated CD34^-^lineage^- ^cells (day 0) and to cells maintained with SCF and Flt3-L either alone or supplemented with IL-21. M = marker. JP = rearrangement involving the Jγ and Cγ segments. The forward primer mapped to the JγP cassette, whereas the reverse primer mapped to the constant (C) region. JP1 = rearrangement involving the JγP1 region and the Cγ segment. The forward primer mapped to the JγP1 cassette, whereas the reverse primer mapped to the constant (C) region. JP2 = rearrangement involving the JγP2 region and the Cγ segment. The forward primer mapped to the JγP2 cassette, whereas the reverse primer mapped to the constant (C) region. J1/2 = rearrangement involving the Jγ1/2 region and the Cγ segment. The forward primer mapped to the Jγ1–2 cassette, whereas the reverse primer mapped to the constant (C) region. L = linker region (no rearrangement). Forward and reverse primers mapped to the spacer region between Vγ9 and Vγ10 segments.

We next aimed to get insights into the expression levels of mRNA encoding for NK-associated transcription factors. The provision of IL-15 and IL-21 to CD34^-^lineage^- ^cells was associated with up-regulated mRNA signals for Bcl-2, GATA-3 and, perhaps more importantly, Id2, a master switch implicated in the commitment of bi-potent foetal thymus T/NK progenitors to NK cells (Figure 4A) [22]. Notably, Bcl-2 induction by IL-21 has also been demonstrated in T cells, leading to their enhanced survival through the activation of the PI-3K signalling pathway [23].

Because T-cell precursors residing in the foetal liver, spleen and blood possess NK lineage potential, we aimed to determine whether NK differentiation in response to IL-15 and IL-21 occurred in association with TCR rearrangement. Previously, we have detected rearranged TCRγ genes in CD34^-^lineage^- ^UCB cells cultured with IL-15 and a stromal cell support [5]. The TCRγ chain gene has 2 constant (C), 5 joining (J) and 14 variable (V) region segments. Most variable region (Vγ) rearrangements occur within the Vγ1–8 subgroup and most joining region (Jγ) rearrangements involve the Jγ1/2 segment [24]. In order to increase TCRγ rearrangement detection rate, we used multiple primer sets specific for C and J regions of the TCRγ chain, as detailed in Materials and Methods. As shown in Figure 4B, CD34^-^lineage^- ^cells cultured with IL-15 and IL-21 harboured un-rearranged TCRγ genes, similar to freshly isolated CD34^-^lineage^- ^cells and to cells maintained with SCF and Flt3-L either alone or supplemented with IL-21. This observation suggests that NK commitment under the experimental conditions here established occurs through a pathway that does not include TCR rearrangement.

### Cytokine/chemokine secretion by IL-21-differentiated NK cells

**Figure 5:**
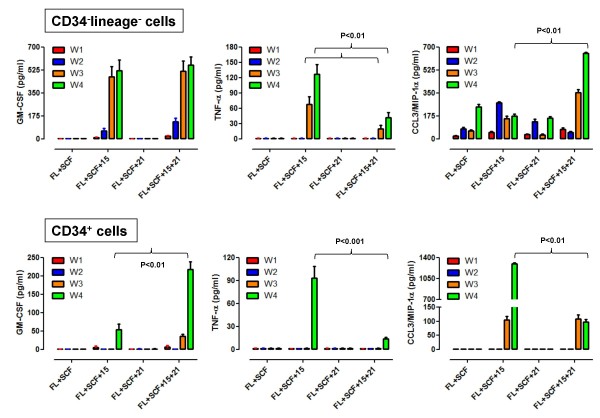
**Cytokine and chemokine release from CD34^-^lineage^- ^cells and CD34^+ ^cells differentiated with IL-15 and IL-21**. The production of GM-CSF, TNF-α and CCL3/MIP-1α in supernatants of CD34^-^lineage^- ^cells and CD34^+ ^cells maintained with SCF+Flt3-L either alone or supplemented with IL-15, IL-21 or the combination of both cytokines was monitored weekly with conventional ELISA. Bars depict mean and standard deviation recorded in 3 independent experiments performed in triplicate. FL = Flt3-L; W = week.

We next determined the ability of IL-15+IL-21-differentiated cells to release IFN-γ, GM-CSF, TNF-α and CCL3/MIP-1α in culture supernatants. IFN-γ was undetectable (< 8 pg/ml) after cell culture in the presence of SCF and Flt3-L supplemented with either IL-15 or IL-21 alone. In sharp contrast, exposure to IL-15 and IL-21 induced a highly significant release of IFN-γ in 4-week old cultures, corresponding to a mean of 1210 ± 245 pg/ml in 10 independent experiments. IFN-γ release by CD34^+ ^HSC matured with IL-15 and IL-21 was superimposable (data not shown). GM-CSF was undetectable in Flt3-L+SCF-differentiated cultures but was released at high levels from week +3 onward in CD34^-^lineage^- ^cultures nurtured with IL-15 alone or with IL-15+IL-21. Conversely, TNF-α was preferentially detected in the supernatants of IL-15-differentiated CD34^-^lineage^- ^cells compared with cells maintained with IL-15 and IL-21. GM-CSF and TNF-α release by CD34^+ ^HSC followed a similar pattern, although the magnitude of cytokine release was significantly lower when compared with CD34^-^lineage^- ^cultures. Finally, measurable CCL3/MIP-1α secretion occurred under any culture condition that we established. However, CCL3/MIP-1α release in response to Flt3-L and SCF was further enhanced by IL-15 alone and, to a greater extent, by IL-15 and IL-21 in combination (Figure 5). Notably, NK cells differentiated with IL-15 from CD34^+ ^HSC released the highest levels of CCL3/MIP-1α. Collectively, these experiments suggested that IL-15+IL-21-differentiated CD34^-^lineage^- ^cells acquired the ability to release soluble factors relevant for NK effector function and NK migration/homing.

### Response of IL-21-differentiated NK cells to maturation stimuli

**Figure 6:**
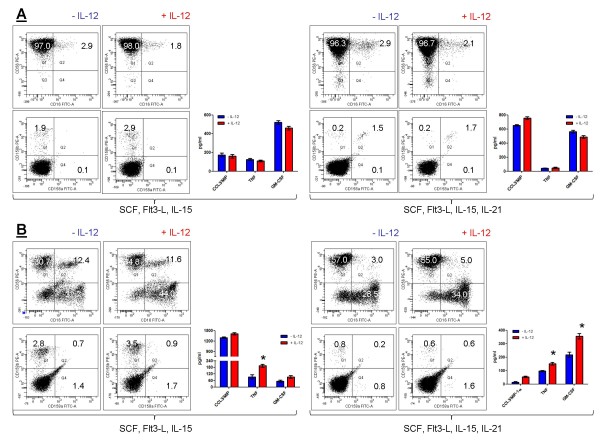
**IL-12-induced maturation of cytokine-differentiated NK cells**. CD34^-^lineage^- ^cells (panel A) and CD34^+ ^cells (panel B) were differentiated with SCF+Flt3-L supplemented with either IL-15 or IL-15+IL-21 for 4 weeks, as already detailed. After further 48 hours of culture with 2 ng/ml IL-12, cells were harvested and used for flow cytometry experiments and for measurements of cytokine/chemokine release. A representative flow cytometry profile out of 4 with similar results is shown. Markers were set according to the proper isotypic control. The blue bars depict cytokine/chemokine release in cultures that were not treated with exogenous IL-12, whereas red bars indicate cytokine/chemokine production in cultures exposed to IL-12. *P < 0.01 compared with cultures established in the absence of IL-12.

We asked whether a cytokine stimulus such as IL-12 could further mature the population of NK cells differentiated with IL-15 and IL-21. We recovered NK cells from 4 week-old cultures of CD34^-^lineage^- ^cells and CD34^+ ^cells, and we exposed them to exogenous IL-12 for additional 48 hours [25]. Cells were collected and used to assess the expression of a restricted panel of NK-cell antigens, whereas supernatants were used to measure TNF-α, MIP-1α and GM-CSF release. As summarised in Figure 6A, no appreciable modification of the expression levels of CD16, CD56 and KIRs occurred after the exposure of IL-15- or IL-15+IL-21-matured NK cells to IL-12. In line with this observation, the release of MIP-1α, TNF-α and GM-CSF was superimposable, irrespective of the provision of IL-12 to cytokine-differentiated NK cells. Whereas IL-12 induced only a modest increase of CD56 expression on CD34-derived NK cells, it significantly enhanced their ability to secrete TNF-α and GM-CSF (Figure 6B). Because equal numbers of cytokine differentiated NK cells were used to detect IL-12-induced maturation, these experiments also indicated that TNF-α and GM-CSF release on a *per cell *basis was remarkably higher in CD34^-^lineage^-^-derived NK cells compared with CD34^+^-derived NK cells.

### Functional assays of NK activity

**Figure 7:**
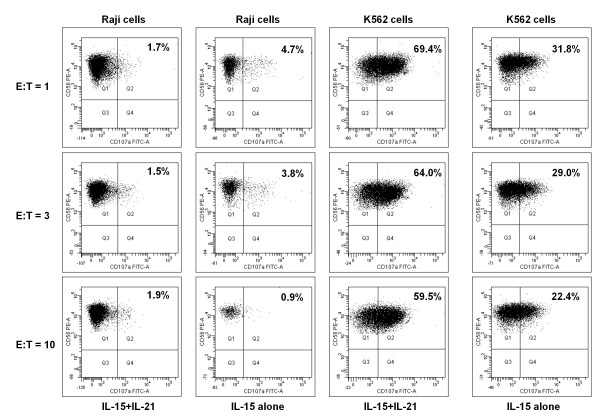
**Degranulation assay with cytokine-differentiated NK cells**. NK cell function was evaluated through flow cytometry monitoring of CD107a expression, following a previously described protocol [26]. CD34^-^lineage^- ^cells initially differentiated with either IL-15 alone or the combination of IL-15 and IL-21 for 4 weeks were further activated for 24 hours with 100 IU/ml IL-2 (R&D Systems), followed by co-culture with NK-sensitive cells (K562) or NK-resistant cells (Raji). The different effector (E)-to-target (T) ratios indicated in the Figure were selected based on previous publications [26]. After 4 hours, cells were harvested, labeled with anti-CD107a and anti-CD56 mAb and analyzed by flow cytometry. The data shown here are representative of 4 independent experiments with similar results. The percentage of CD107a^+^CD56^+ ^cells is indicated.

CD34^-^lineage^- ^cells cultured with IL-15 and IL-21 for 4 weeks were assayed for NK activity against NK-sensitive tumour cell targets. To this end, cytokine-differentiated cells were first activated with IL-2 and then co-cultured at escalating effector-to-target ratios with either NK-resistant Raji cells or NK-sensitive K562 cells for 4 hours. The expression of CD107a, a lysosomal protein whose up-regulation reflects exocytosis of secretory granules, on CD56^+ ^NK cells was monitored by flow cytometry [26]. Control cultures were established with NK cells differentiated from UCB CD34^+ ^cells under the same culture conditions as those applied to CD34^-^lineage^- ^cells. As shown in Figure 7, minor degranulation occurred when NK-resistant Raji tumour cells were plated in co-culture with NK cells derived from either CD34^-^lineage^- ^cells or CD34^+ ^HSC (data not shown). Conversely, an average 65 ± 11% of CD56^+ ^NK cells stained positively for CD107a in co-cultures established with NK-sensitive K562 cells and NK cells differentiated from CD34^-^lineage^- ^cells with IL-15 plus IL-21. Notably, NK cell degranulation occurred at significantly lower levels in co-cultures containing K562 cells and IL-15-differentiated CD34^-^lineage^- ^cells (mean percentage of CD107a^+^CD56^+ ^NK cells equal to 35 ± 6 at E:T ratio = 1; p < 0.01), suggesting that IL-15 and IL-21 exerted synergistic effects on NK activity. Finally, NK cells differentiated from CD34^+ ^HSC with either IL-15 alone or IL-15+IL-21 manifested a similar cytotoxic activity to that of cytokine-differentiated CD34^-^lineage^- ^cells (data not shown).

## Discussion

NK cells are important effectors of the innate immune system and exhibit cytolytic activity against infectious agents and tumour cells. Although our knowledge of NK-cell developmental intermediates remains limited, advances have recently led to a better definition of appropriate culture conditions for the *in vitro *generation of mouse and human NK cells from foetal thymus [27], foetal liver [28], UCB [15] and bone marrow HSC [29]. In early studies, NK-cell development from purified HSC was shown to be stromal-cell dependent [30]. It has later been demonstrated that the stromal-cell requirements may be replaced by the provision of early-acting cytokines such as SCF, Flt3-L and IL-7 to the cultures [31]. In particular, SCF and Flt3-L directly induce the expression of IL-2 receptor-β chain on HSC, thereby rendering them susceptible to the NK-cell commitment induced by IL-15 [31]. Prolonged culture of CD34^+ ^HSC with IL-15 in the absence of stromal cells can generate pseudo-mature lytic NK cells, e.g., cells expressing markers of mature NK cells (NK1.1 and DX5 in mice, CD56 in humans, CD94-NKG2 receptors in both species) but not Ly49 receptors or KIR [29,32]. More recently, UCB CD34^+ ^cells have been differentiated along the NK lineage with Flt3-L, IL-15, IL-21, and hydrocortisone but in the absence of any stromal cell support [16]. In addition, IL-21 may synergize with IL-7, IL-15, SCF, Flt3-L and serum supplementation in promoting the generation of NK cells from UCB CD34^+ ^cells [15].

The antitumor activity of IL-21 has been demonstrated in murine experimental models where direct effects of IL-21 on NK cells were responsible for tumour suppression [33]. In addition, the ability of IL-21 to promote long-lasting CD8^+ ^T-cell-dependent tumour responses has been shown in athymic mice with intraperitoneal or subcutaneous tumours [34,35]. IL-21 may also augment human T-cell proliferation driven by polyclonal activation or by a peptide in the absence of other stimuli and may increase CD8^+ ^T-cell production of IFN-γ induced by IL-15 [36]. The aforementioned *in vitro *and pre-clinical findings have prompted the evaluation of IL-21 as immunotherapy for patients with metastatic melanoma and renal cell carcinoma [37,38]. These studies have clearly shown that repeated cycles of IL-21 are well tolerated as an outpatient regimen, thus encouraging further development of IL-21 as an immunotherapy for cancer.

Early studies of UCB transplantation for haematological malignancies have demonstrated an impaired rate and quality of immune reconstitution, which may be associated with an increased rate of infectious complications, particularly at early time points after transplantation [39]. These clinical observations reinforce the need for novel cell-based therapeutic approaches to overcome the potentially life-threatening infections, including those attributable to a delayed anti-CMV immunity [40].

We provide evidence that IL-21 favours the NK cell differentiation of CD34^-^lineage^- ^UCB cells in cooperation with IL-15 and in the absence of stromal cell support and serum or hydrocortisone supplementation. The combination of IL-15 and IL-21 displayed a remarkable ability to promote the outgrowth of CD34^-^lineage^- ^cells into NK cells, at variance with IL-21 alone. This is backed by previous observations indicating that the γc-dependent cytokines IL-15 and IL-21 may integrate their signalling and synergise in regulating CD8^+ ^T-cell expansion and function [13]. Using murine mature NK cells, Kasaian et al. [41] have shown that IL-21 may constrain the IL-15-induced expansion of NK cells *in vitro*, although their activation status remains unaffected, underpinning the concept that IL-21 may exert diverging effects on murine as opposed to human NK cells. It has also been demonstrated that low doses of IL-21 increase the proliferative response of murine NK cells to either IL-2 or IL-15, whereas high doses of IL-21 may exert an inhibitory effect on NK cell outgrowth [42]. In our study, IL-21 significantly inhibited the proliferation of CD34^+ ^cells induced by SCF and Flt3-L, suggesting that IL-21 may also exert opposite effects on HSC proliferation depending on the concomitant cytokine stimulus that is applied.

It should be emphasised that CD34^+ ^HSC differentiated under the same cytokine conditions expanded more vigorously than their CD34^-^lineage^- ^counterpart. Not unexpectedly, IL-21 promoted the activation of Stat1 and Stat3, but not Stat5 protein, in CD34^-^lineage^- ^cells. NK cells generated *in vitro *with IL-15 and IL-21 acquired a CD56^+^CD16^-/+ ^phenotype which differs from the phenotype that we previously observed using Flt3-L, SCF, IL-15 and a stromal feeder layer, where NK progenitor cells stained negatively for CD16 [5]. The percentage of CD56-expressing CD34^-^lineage^- ^cells was significantly higher compared to that in cultures of CD34^+ ^HSC, indicating that the former HSC subset has the ability to give origin to a virtually pure NK cell population when confronted with IL-15 and IL-21 *in vitro*. The natural cytotoxicity receptor NKp46 and the NKG2D antigen were strongly up-regulated on CD34^-^lineage^- ^cells cultured with the combination of IL-15 and IL-21. The activating receptor NKG2D, whose ligands are frequently over-expressed in tumours from multiple origins [43], could be detected at very low levels in cultures performed with IL-15 alone. Conversely, NKG2D expression levels significantly increased as a result of combined treatment with IL-15 and IL-21, suggesting that the NK cell populations obtained under these culture conditions may represent suitable effectors for cell-based anti-tumour therapeutic approaches. From a molecular standpoint, IL-15 and IL-21 induced mRNA signals for Bcl-2, GATA-3 and for the NK cell-associated transcription factor Id2. Interestingly, NK cell differentiation occurred through a pathway that does not involve TCR rearrangement, indicating that the NK intermediates originating from UCB CD34^-^lineage^- ^cells differ from previously described bi-potent NK/T cells [27,28].

Considerable release of IFN-γ only occurred in 4-week old cultures of CD34^-^lineage^- ^cells maintained with IL-15 and IL-21. Conversely, GM-CSF and TNF-α could be detected in supernatants of cultures maintained with either IL-15 alone or IL-15 plus IL-21. Significant secretion of TNF-α could be measured preferentially in cultures stimulated with IL-15 alone. These findings are in good agreement with previous reports on IL-21-induced changes of cytokine secretion by UCB-derived CD34^+ ^HSC [16]. In the latter study, IL-21 increased IL-10 and GM-CSF production but lessened TNF-α release after 4-week culturing in the presence of hydrocortisone, Flt3-L and IL-15. CCL3/MIP-1α production occurred under any culture condition herein examined, although the highest production could be documented after challenge with IL-15 and IL-21. The robust GM-CSF and IFN-γ release induced by IL-15 and IL-21 in combination suggests that cytokine-differentiated NK cells may retain the ability to mount effective anti-viral and anti-tumour responses. The significant secretion of CCL3/MIP-1α, a chemokine implicated in the selective mobilization of NK cells from the bone marrow compartment into the peripheral blood [44], implies that cytokine-matured NK cells may provide *in vivo *signals contributing to the regulation of NK homing, retention and migration.

The NK cell populations differentiated with IL-15 and IL-21 were resistant to further maturation with IL-12, as evaluated both in terms of surface membrane phenotype and in terms of cytokine/chemokine release. Specifically, the expression levels of CD16, CD56 and KIR were similar irrespective of the provision of exogenous IL-12. Similarly, CCL3/MIP-1α, TNF-α and GM-CSF release were superimposable in cultures of IL-15+IL-21-differentiated cells that were either stimulated with IL-12 or left untouched.

Finally, the NK cells differentiated with IL-15 and IL-21 underwent exocytosis of secretory granules, as measured by a flow cytometry-based CD107a degranulation assay, upon co-culturing for 4 hours with NK-sensitive tumour cell targets, indicating the acquisition of cytolytic potential. However, the extent of NK granule exocytosis was comparable to that measured in co-cultures of K562 cells and NK cells differentiated from CD34^+ ^HSC with the combination of IL-15 and IL-21.

## Conclusion

This study suggests that considerable numbers of highly pure, lytic CD56^+^CD16^-/+ ^NK cells for adoptive immunotherapy can be obtained from UCB CD34^-^lineage^- ^cells using a serum-free, feeder cell-free culture system. From a qualitative standpoint, these NK populations differ from those differentiated from UCB CD34^+ ^HSC insofar they express high levels of the activating receptor NKG2D, release high quantities of IFN-γ, GM-CSF and TNF-α and are resistant to maturation with IL-12. The findings highlighted herein also shed some light into the developmental intermediates of NK cells that can be differentiated after the exposure of CD34^-^lineage^- ^cells to IL-21.

## Methods

### Isolation and culture of human CD34^-^lineage^- ^cells

CD34^-^lineage^- ^cells were purified from UCB samples collected after full-term delivery from consented donors [5]. All investigations were approved by local Human Research Committees. Briefly, UCB mononuclear cells were obtained by Ficoll-Hypaque density gradient centrifugation and CD34^+ ^cells were separated using the MACS system (Miltenyi Biotec GmbH, Bergisch Gladbach, Germany). The CD34^- ^fraction was further depleted of lineage^+ ^cells, as previously detailed [5]. Freshly isolated CD34^-^lineage^- ^cells comprised 0.22 ± 0.09% of UCB mononuclear cells (number of samples analyzed = 8) and stained negatively for lymphoid/NK cell markers (not shown and previously published data [5]). CD34^-^lineage^- ^cells were cultured with MyeloCult™ H5100 (Stem Cell Technologies, Vancouver, Canada) supplemented with 10^-6^M hydrocortisone (Sigma Aldrich, Milan, Italy), 20 ng/ml SCF and 20 ng/ml Flt3-L (R&D Systems, Oxon, Cambridge, United Kingdom). IL-15 (50 ng/ml) and/or IL-21 (20 ng/ml; R&D Systems) were added to SCF/Flt3-L-containing cultures as specified in the Figure legends. In selected experiments, 2 ng/ml IL-12 was provided to the NK-cell cultures for 2 days in the attempt to induce complete phenotypic and functional maturation [45].

### Flow cytometry

Cells were incubated for 30 minutes at 4°C with fluorochrome-conjugated monoclonal antibodies (mAb) to CD16, CD34, CD56 (BD Biosciences, Mountain View, CA), CD94, CD158a, CD158b (Pharmingen, San Diego, CA), NKG2A, IL-21 receptor (R&D Systems), CD45 (Caltag Laboratories, Burlingame, CA), CD94, NKp46 (CD335), NKG2D (CD314; Beckman Coulter, Milan, Italy). Isotype-matched, fluorochrome-conjugated mAb from the same manufacturers were used to control for background fluorescence. Cells were run through a FACS Canto^® ^flow cytometer (BD) with standard equipment [46].

### CD107a degranulation assay

NK cells were activated with 100 IU/ml IL-2 for 48 hours. After washings with PBS, NK cells were co-cultured for 4 hours with either K562 (NK-sensitive) or Raji cells (NK-resistant) at 1:1, 1:3 and 1:10 effector-to-target (E:T) ratio, as previously published [26]. Thereafter, cells were labelled with PE-conjugated anti-CD56 and FITC-conjugated anti-CD107a antibodies (both from BD Biosciences) for 20 minutes at 4°C, followed by flow cytometry analysis. Isotype-matched antibodies from the same manufacturer were used to assess background fluorescence.

### Reverse transcriptase polymerase chain reaction (RT-PCR)

Details on RNA extraction were previously published [47,48]. One μg of total RNA was reverse-transcribed with 25 U of Moloney murine leukaemia virus reverse transcriptase (PE Applied Biosystem, Foster City, CA) at 42°C for 30 minutes in the presence of random hexamers. Two μl of cDNA products were amplified with 1 U of AmpliTaq Gold (PE Applied Biosystem) in the presence of primers specific for the RNA of interest [49]. Amplification of human Id2 mRNA (GI 464183) was achieved by 29 cycles of 45 seconds at 56°C and 1 minute at 72°C, using the following primers (M-Medical, Florence, Italy): 5'-GATATCAGCATCCTGTCCTT-3' and 5'-CATTCAGTAGGCTTGTGTGA-3'.

### Analysis of TCRγ rearrangement

The extraction of genomic DNA was performed with the QIAamp DNA Blood Mini Kit (Qiagen, Milan, Italy). The analysis of TCRγ rearrangement was conducted with primers listed in Table 2 (GI 28436398). The reaction mixtures contained 10× PCR buffer, 0,8 μM of each dNTP, 3 mM MgCl_2_, 0.2 mM of each primer, 5% DMSO and 1,25 U of Amplitaq Gold (PE Applied Biosystem). Four separate reaction mixes were prepared with each one containing a Jγ direct primer coupled with a reverse primer specific for the constant region. Control PCR were run with two reaction mixes, one containing a direct and reverse primer mapping in the constant region segment and the other containing a direct and reverse primer mapping in the TCR linker region (Table 2). Semi-nested PCR were performed using internal reverse primers.

**Table 2:** Primers used to detect TCRγ rearrangement in cytokine-differentiated CD34^-^lineage^- ^cells.

***Primer***	***Sequence (5'-3')***	***Region***	***Orientation***
TCRL	TCCTACTGATCACTGTGCTG	Linker (L) region	F
TCRL-IR	GGCCTTAAGTTGGCTGCATC		R
TCRL-ER	TGTTCTGTTGGAGATGGGAC		R
			
TCR 1/2	CGCATATCATCTGTCAGAAC	Constant (C) region	F
TCR 1/2IR	TACCACGTGTCACGTTGCTA		R
TCR 1/2ER	TGTCAACAATGAACCCGTTG		R
			
JγPF	AATCAAGGTATTTGGTCCCG	J cassette	F
JγP1F	TACCACTGGTTGGTTCAAGA		F
JγP1/2F	AAAGGGACTAGGCTCATAGT		F
Jγ1/2F	AACAACACTTGTTGTCACAG		F

Abbreviations: TCRL = TCR linker; TCRL-I = TCR linker internal; TCRL-E = TCR linker external; ER = external reverse; IR = internal reverse; F = forward; R = reverse.

### Measurement of cytokine and chemokine release

The production of IFN-γ, GM-CSF, TNF-α and MIP-1α by CD34^-^lineage^- ^cells and CD34^+ ^cells differentiated with IL-15 and IL-21, either alone or in combination, was investigated after their activation with 10 μg/ml PHA for 18 hours. Culture supernatants were harvested and IFN-γ, GM-CSF, TNF-α and MIP-1α were quantitated using commercially available ELISA (R&D Systems, Oxon, Cambridge, UK). The minimum detectable doses, as reported by the manufacturer, were as follows: 8 pg/ml IFN-γ; < 3 pg/ml GM-CSF; 1.6 pg/ml TNF-α; < 10 pg/ml CCL3/MIP-1α.

### Detection of Stat1, Stat3 and Stat5 activation

The relative amount of Stat1 (Tyr701), Stat3 (Tyr705) and Stat5 (Tyr694) phosphorylation after IL-21 provision to CD34^-^lineage^- ^cells was determined with phospho-Stat-specific mAb, following the manufacturer's instructions (RayBio^® ^Cell-Based STAT ELISA Sampler Kit; RayBiotech Inc., Norcross, GA). Briefly, 20–30 × 10^3 ^CD34^-^lineage^- ^cells were seeded in a 96-well plate and incubated overnight at 37°C, 5% CO_2_. IL-21 was then added to the wells at 20 ng/ml (final concentration) for up to 4 hours, as detailed in the Figure legends. After cytokine challenge, cells were fixed and extensively washed with the appropriate buffer solution, and then incubated with antibodies directed against phosphorylated Stat proteins for 2 hours at room temperature. After further washings, cells were incubated with HRP-conjugated anti-mouse IgG for 1 hour and treated with 3,3',5,5'-tetramethylbenzidine (TMB) substrate solution for 30 minutes at room temperature. Optical density (OD) was immediately measured at 450 nm.

### Quantitative PCR

A real-time quantitative RT-PCR for the genes of interest (GATA-3, IDH2, BCL-2, CD34, GAPDH; Table 3) was performed with the iCycler iQ system (Bio-Rad, Hercules, CA) on RNA samples obtained from freshly isolated and cytokine-conditioned cells. Complementary DNA (cDNA) was prepared starting from 1 μg of total RNA, after DNase I treatment (Amersham Pharmacia biotech, Buckinghamshire, UK) using the iScrypt cDNA Synthesis Kit (containing RNase H + MMLV reverse transcriptase random primers and 5× reaction mixture (Bio-Rad) according to the manufacturer's instructions. Reactions were conducted in the PTC-0200 DNA Engine (MJ RESEARCH). Amplification was carried out in a total volume of 25 μl containing 0.3 μM of each specific primer, 12,5 μl 2× SYBR Green Master Mix (Bio-Rad) (containing 100 mM KCl, 40 mM Tris-HCl, pH8.4, 0.4 mM of each dNTP, iTaq DNA Polymerase, 6 mM MgCl_2_, SYBR Green I, 20 nM fluorescein, stabilizers) and 2 μl of diluted cDNA. The PCR reactions were cycled starting with a 3-minute template denaturation step at 95°C followed by 40 cycles of 15 seconds at 95°C and 1 minute at 60°C. Complementary DNA from heart tissue cells was used as control in each set of reactions. Standard curves were generated using a serial dilution of the initial amount of control cDNA to determine the range of template concentration, which showed a good linearity (0,998–0,999) and efficiency (>90%) for the different reactions. Melt curves of the reaction products were also generated to assess the specificity of the measured fluorescence. Samples were run in triplicate and the mean of threshold cycles (*C*_*t*_) for each specimen was used to obtain the fold change of GATA-3, IDH2, BCL-2, and CD34 expression level, using the following equation:

**Table 3:** Primers used to detect gene expression by qPCR in cytokine-differentiated CD34^-^lineage^- ^cells.

***Primer***	***Sequence (5'-3')***	***Orientation***
IDH2	ACTACATCAGGGACCTTCAG	F
IDH2	TATAAGGATGATCTAGTGGTCG	R
BCL-2	AGGAAACTTGACAGAGGATCATGC	F
BCL-2	CGGATCTTTATTTCATGAGGCACG	R
GATA-3	AGGAGGAATGCCAATGGGGAC	F
GATA-3	TCGGTTTCTGGTCTGGATGCC	R
CD34	TCTTGACAACAACGGTACTGCTAC	F
CD34	GCTGGTACTTCCAAGGGTACTAGG	R
GAPDH	CCTGACCTGCCGTCTAGAAA	F
GAPDH	CTCAGTGTAGCCCAGGATGC	R



where Δ*Ct *= *C*_*t *specific gene _-*C*_*t *GAPDH _and Δ(Δ*Ct) *= Δ*C*_*t *specimen _- Δ*C*_*t *control_. A sample with a 1-fold change represents a sample with the same expression level as the reference control for a target gene. Calculations were performed with the Excel spreadsheet RelQuant (Bio-Rad, last update January 2004). Primer sets were designed using the Beacon Design Software (Version 3) and the sequences available in the GeneBank™ data base. The specific oligonucleotide primer sequences are detailed in Table 3.

### Statistical methods

The approximation of population distribution to normality was tested preliminarily using statistics for kurtosis and symmetry. Data were presented as mean ± SD and comparisons were performed with the Student's *t *test for paired or unpaired data or with the analysis of variance, as appropriate. The criterion for statistical significance was defined as *p *= .05.

## Authors' contributions

GB carried out the experiments and participated in the design of the study. AM, AP and MC carried out the experiments. GS participated in the design of the study. LP participated in the design of the study. SR participated in the design of the study, carried out the experiments, performed the statistical analysis and drafted the manuscript. All authors read and approved the final manuscript.
